# An intermolecular binding mechanism involving multiple LysM domains mediates carbohydrate recognition by an endopeptidase

**DOI:** 10.1107/S139900471402793X

**Published:** 2015-02-26

**Authors:** Jaslyn E. M. M. Wong, Søren Roi Midtgaard, Kira Gysel, Mikkel B. Thygesen, Kasper K. Sørensen, Knud J. Jensen, Jens Stougaard, Søren Thirup, Mickaël Blaise

**Affiliations:** a Centre for Carbohydrate Recognition and Signalling, Department of Molecular Biology and Genetics, Aarhus University, Gustav Wieds Vej 10C, 8000 Aarhus, Denmark; b Niels Bohr Institute, Faculty of Science, University of Copenhagen, Universitetsparken 5, 2100 Copenhagen, Denmark; c Centre for Carbohydrate Recognition and Signalling, Department of Chemistry, Faculty of Science, University of Copenhagen, Thorvaldsensvej 40, 1871 Frederiksberg C, Denmark

**Keywords:** d,l-endopeptidase, LysM domains

## Abstract

LysM domains, which are frequently present as repetitive entities in both bacterial and plant proteins, are known to interact with carbohydrates containing *N*-acetylglucosamine (GlcNAc) moieties, such as chitin and peptidoglycan. In bacteria, the functional significance of the involvement of multiple LysM domains in substrate binding has so far lacked support from high-resolution structures of ligand-bound complexes. Here, a structural study of the *Thermus thermophilus* NlpC/P60 endopeptidase containing two LysM domains is presented. The crystal structure and small-angle X-ray scattering solution studies of this endopeptidase revealed the presence of a homodimer. The structure of the two LysM domains co-crystallized with *N*-acetyl-chitohexaose revealed a new intermolecular binding mode that may explain the differential interaction between LysM domains and short or long chitin oligomers. By combining the structural information with the three-dimensional model of peptidoglycan, a model suggesting how protein dimerization enhances the recognition of peptidoglycan is proposed.

## Abstract 2

The crystal and solution structures of the *T. thermophilus* NlpC/P60 d,l-endopeptidase as well as the co-crystal structure of its N-terminal LysM domains bound to chitohexaose allow a proposal to be made regarding how the enzyme recognizes peptidoglycan.

## 1. Introduction   

Most bacteria are protected from their environment by a rigid cell wall containing peptidoglycan (PGN), a disaccharide polymer of *N*-acetylglucosamine (GlcNAc) and *N*-acetyl­muramic acid (MurNAc) (Dworkin, 2014 ▶). A three-dimensional PGN network is formed owing to cross-linking of the peptide stems attached to MurNAc (Meroueh *et al.*, 2006 ▶; Kim *et al.*, 2015 ▶). The composition of the peptide stems differs among species, but is usually made up of three to five amino acids that include noncanonical d-amino acids. The greatest variation lies in the third amino acid, which is often α-l,∊-d-diaminopimelic acid (*meso*-DAP) or l-lysine; l-ornithine has also been reported (Vollmer, Blanot *et al.*, 2008 ▶; Quintela *et al.*, 1995 ▶). Although PGN is very rigid, it has also been shown to be sufficiently dynamic to allow bacterial to elongate and separate during cell division (Typas *et al.*, 2012 ▶). During these dynamic phases, PGN is remodelled, and the balance between PGN synthesis and hydrolysis has to be tightly controlled to ensure bacterial survival (Egan & Vollmer, 2013 ▶).

Numerous enzymes termed autolysins are involved in PGN remodelling. Glycosidases, such as muraminidases and glucosaminidases, hydrolyze glycosidic bonds between carbo­hydrate units, while peptidases, such as amidases, l,d-endopeptidases, d,l-endopeptidases, l,d-carboxypeptidases and d,d-carboxypeptidases, hydrolyze amide bonds of the peptide stem at specific positions (Vollmer, Joris *et al.*, 2008 ▶). d,l-Endopeptidases belonging to the papain-like peptidase superfamily possess an NlpC/P60 domain which is responsible for their catalytic activity (Anantharaman & Aravind, 2003 ▶). This domain is commonly associated with PGN-binding domains such as the SH3b domain, choline-binding domain or lysin motif (LysM) (Anantharaman & Aravind, 2003 ▶; Xu *et al.*, 2009 ▶). These domains are assumed to assist in anchoring the protein to the cell wall. However, many of the crystallographic or NMR structures of NlpC/P60 endopeptidases deposited in the Protein Data Bank (PDB) contain only the catalytic domains. To date, only the structures of the NlpC/P60 proteins from the cyanobacteria *Anabaena variabilis*, *Nostoc punctiforme* and *Bacillus cereus* have been solved with their N-terminal SH3b domains (Xu *et al.*, 2009 ▶, 2010 ▶). In addition, the structure of the NlpC/P60-related amidase of AmiA from *Bacteroides uniformis* has recently been solved in complex with GlcNAc and GlcNAc-1,6-anhydro-MurNAc, providing insights into the substrate recognition and specificity of the enzyme (Xu *et al.*, 2014 ▶). However, no NlpC/P60 structures associated with the choline-binding domain or LysM domains have been solved, and there remains a lack of NlpC/P60 structures that have been solved in complex with PGN fragments containing carbohydrate units. As such, there is a limited understanding of how these enzymes anchor onto PGN and how the substrates are delivered to the catalytic domain.

We and others have shown that the LysM domain does indeed mediate recognition of PGN (Visweswaran *et al.*, 2013 ▶, 2014 ▶; Wong *et al.*, 2014 ▶; Maolanon *et al.*, 2014 ▶; Frankel & Schneewind, 2012 ▶; Mesnage *et al.*, 2014 ▶; Schanda *et al.*, 2014 ▶). Our study of the multiple LysM-containing protein CwlS from *B. subtilis* also demonstrated that the NlpC/P60 endopeptidase displays an affinity towards PGN in the micromolar range. This modest affinity suggests that the multiple LysM modules present in the N-terminus of NlpC/P60 proteins may be crucial for anchoring the proteins to PGN and consequently for their hydrolytic function (Wong *et al.*, 2014 ▶). Recent biochemical approaches have suggested that multiple LysM domains cooperate to enhance binding to GlcNAc polymers. However, none of these studies were able to conclude whether this affinity enhancement was owing to the fact that each LysM domain can bind a carbohydrate molecule or to the fact that several LysM domains can bind to the same carbohydrate molecule (Wong *et al.*, 2014 ▶; Mesnage *et al.*, 2014 ▶), or a combination of both.

The crystal structure of the fungal Ecp6 chitin-scavenger protein containing three LysM domains has shown that chitin is recognized at the interface of two intrachain LysM domains (Sánchez-Vallet *et al.*, 2013 ▶). Dimerization of plant AtCERK1 receptors on long chitin polymers has also been demonstrated and has been suggested to be important for immune signalling (Liu *et al.*, 2012 ▶). Recently, a ‘sandwich-type’ dimerization mode has also been proposed for the recognition of chitin by the CEBiP–OsCERK1 receptor complex that is involved in plant immunity (Hayafune *et al.*, 2014 ▶). However, no structural information has supported this sandwich model of intermolecular dimerization.

**Figure 1:**
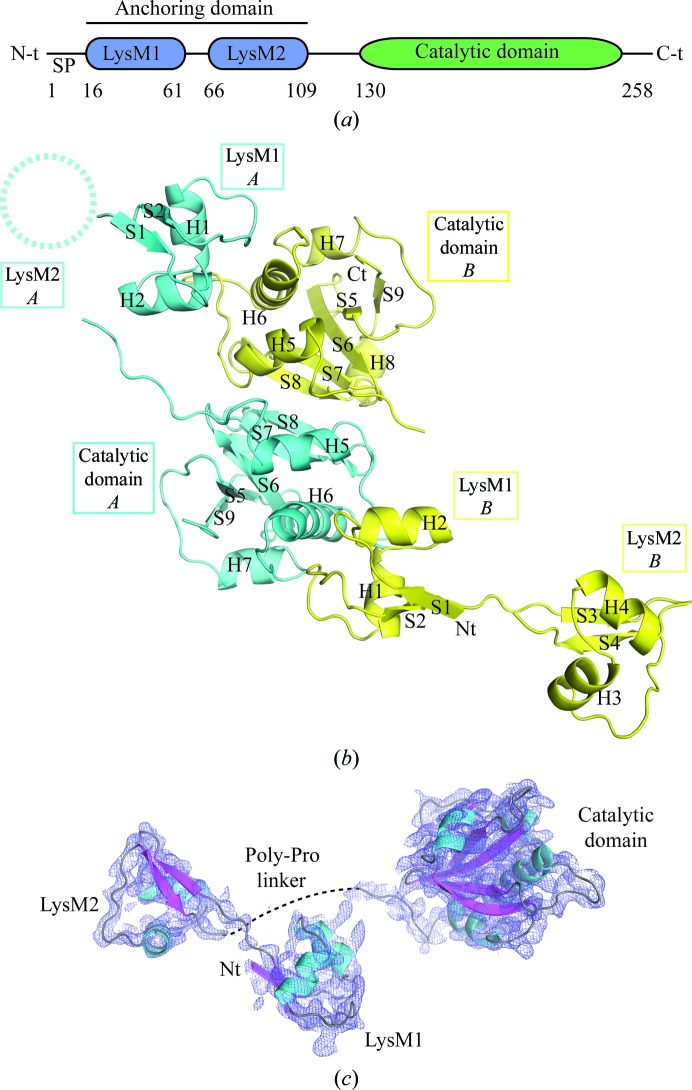
Overall structure of P60_tth. (*a*) Scheme of P60_tth: the two LysM domains forming the anchoring domain are represented in blue and the catalytic domain is coloured green. SP, signal peptide. (*b*) Composition of the asymmetric unit. The two molecules composing the asymmetric unit are represented in cyan and yellow in the cartoon. The dashed circle indicates the LysM domain for which we could not see any electron density. Strands and helices are denoted S and H, respectively, followed by their number. (*c*) Structure of the P60_tth monomer represented as a cartoon with strands and helices coloured cyan and magenta, respectively. The dashed line represents the missing polyproline linker. The final 2*F*_o_ − *F*_c_ electron-density map displayed as a blue mesh is contoured at the 1σ level.

In this study, we unravel the crystallographic and solution structure of TTHA0266 (renamed P60_tth), an NlpC/P60 d,l-endopeptidase from *Thermus thermophilus* that possesses an N-terminal PGN-anchoring domain made up of two LysM domains (Fig. 1 ▶ *a*). We also report a co-crystal structure of P60_2LysM (P60_tth with no catalytic domain) bound to *N*-acetyl-chitohexaose (henceforth referred to as chitohexaose), which sheds light on how LysM domains cooperate to bind long chitin/PGN polymers. Based on these high-resolution structural investigations, we propose a model describing how LysM domains may help to anchor the catalytic domains of the d,l-endopeptidase onto PGN.

## 2. Materials and methods   

### 2.1. Gene cloning, protein expression and purification   

The *TTHA0266* gene was cloned and the P60_tth protein was expressed and purified as described previously (Wong & Blaise, 2013 ▶). The gene was cloned in frame with a Trx tag, a His tag and an S-tag into pET-32 Ek/LIC expression vector, which served as a template for generating the truncation mutants P60_2LysM (no catalytic domain), P60_1LysM (no N-terminal LysM domain) and P60_cata (catalytic domain alone) using the following primers: for P60_2LysM, the reverse primer GAGGAGAAGCCCGGTTACGCCTCGCCCTCTTCGGGAAGCCTCAGGACCTGCCCCACCTTG; for P60_1LysM, the forward primer GACGACGACAAGATG**GAGAATCTGTACTTCCAGGGA**TCGAGGGAAAGGACCCACGTGGTGGCCCCGGGGGACACC; and for P60_cata, the forward primer GACGACGACAAGATG**GAGAATCTG** **TACTTCCAGGGA**GAAAGCCCCCTCCTCCGGGCCGTCCTCCGCTACCTGGGG. The sequence in bold encodes the *Tobacco etch virus* (TEV) protease cleavage site that was introduced to facilitate the removal of the affinity tags during the protein purification process. The genes for the aforementioned truncation mutants were all cloned into the pET-32 Ek/LIC vector (Novagen). The P60_tth_LysM1_mut and P60_tth_LysM2_mut binding mutants in the pET-44 and pET-32 Ek/LIC vectors (Novagen), respectively, were generated using the QuikChange Lightning Site-Directed Mutagenesis Kit (Agilent Technologies) according to the manufacturer’s instructions. The pET-44 Ek/LIC vector encodes a His tag, a Nus tag, a His tag and an S-tag at the N-terminus. All mutants were produced and purified using the same procedures as used for the wild-type protein. Briefly, the recombinant proteins were produced in *Escherichia coli* BL21 Rosetta 2 (DE3) competent cells (Novagen), which were lyzed by sonication. The purification steps included an initial round of nickel-affinity chromatography (IMAC), TEV protease cleavage, a second round of IMAC and size-exclusion chromatography using a Superdex 75 10/300 GL column (GE Healthcare). For P60_cata, thrombin cleavage was performed after the first round of IMAC. For the P60_tth_LysM1_mut construct, an additional anion-exchange chromatography step was introduced after the second round of IMAC to separate the cleaved tags from the protein; this was performed using a 1 ml HiTrap DEAE FF column (GE Healthcare). All purification steps were performed at 4°C and all proteins were at least 95% pure after the final step of purification.

### 2.2. Crystallization and structure determination   

The full-length protein structure was solved using selenomethionine-derivative crystals as described previously (Wong & Blaise, 2013 ▶). Briefly, the selenomethionine-derivative protein was crystallized at 19°C in hanging drops composed of 1 µl protein solution at 24 mg ml^−1^ and 1 µl reservoir solution consisting of 0.1 *M* sodium citrate pH 5.5, 16%(*w*/*v*) PEG 4000, 15%(*v*/*v*) 2-propanol equilibrated against 500 µl reservoir solution. Crystals were soaked briefly in cryoprotectant solution consisting of 0.1 *M* sodium citrate pH 5.5, 16%(*w*/*v*) PEG 4000 and 20% ethylene glycol prior to being cryocooled in liquid nitrogen. Data collection was performed at a wavelength of 0.978 Å on the I911-3 beamline at the MAX-lab synchrotron, Lund, Sweden (Ursby *et al.*, 2013 ▶) as described previously (Wong & Blaise, 2013 ▶). The structure was solved by single-wavelength anomalous dispersion (SAD) phasing (Hendrickson & Teeter, 1981 ▶) as described in Wong & Blaise (2013 ▶).

P60_2LysM was crystallized at 19°C in conditions consisting of 28%(*w*/*v*) PEG MME 2000 and 0.1 *M* potassium thio­cyanate. Sitting drops set up by adding 1 µl reservoir solution to 1 µl 50 mg ml^−1^ protein solution were equilibrated against 500 µl reservoir solution. The crystal was soaked briefly in mother liquor containing 34%(*w*/*v*) PEG MME 2000 prior to cryocooling in liquid nitrogen. Data were collected on the I911-3 beamline at MAX-lab. The data set consisted of 200 frames collected with 1° oscillation range, 5 s exposure time, a wavelength of 0.98 Å and a crystal-to-detector distance of 204.8 mm.

The structure of P60_2LysM bound to chitohexaose was obtained by co-crystallizing the two LysM domains with chitohexaose (Megazyme) at a protein:sugar molar ratio of 1:2 by dissolving the carbohydrate powder directly in the protein solution and incubating it overnight on ice. The complex was crystallized at 19°C in conditions consisting of 1.6 *M* ammonium sulfate, 0.1 *M* MES pH 6.5 and 5%(*v*/*v*) 1,4-dioxane. Hanging drops set up by adding 0.5 µl reservoir solution to 0.5 µl protein solution at 32 mg ml^−1^ were equilibrated against 500 µl reservoir solution. The crystal was soaked briefly in a solution consisting of 1.6 *M* ammonium sulfate, 0.1 *M* MES pH 6.5, 20%(*v*/*v*) 1,4-dioxane and 5%(*v*/*v*) glycerol prior to cryocooling in liquid nitrogen. Data were collected on the I911-2 beamline at MAX-lab (Mammen *et al.*, 2002 ▶). The data set consisted of 200 frames collected with 1° oscillation range, 5.2 s exposure time, a wavelength of 1.04 Å and a crystal-to-detector distance of 100 mm.

All three structures were refined with the *PHENIX* package (Adams *et al.*, 2011 ▶) and model building was performed with *Coot* (Emsley *et al.*, 2010 ▶). The quality of the three structures was checked with *MolProbity* (Chen *et al.*, 2010 ▶), giving the following core/allowed statistics for the Ramachandran plot: 95.3/4.7% for the full-length structure, 97.6/2.4% for the P60_2LysM–chitohexaose structure and 97.4/2.6% for the P60_2LysM structure.

### 2.3. Size-exclusion chromatography   

A calibration curve was obtained using the Gel Filtration Markers Kit for Protein Molecular Weights 6 500–66 000 Da (Sigma–Aldrich) by plotting the partition coefficient *K*_av_ against the logarithm of the molecular weight of the standard proteins. Proteins were loaded onto a Superdex 75 10/300 GL column (GE Healthcare) and eluted with buffer consisting of 50 m*M* Tris–HCl pH 8, 200 m*M* NaCl and 5 m*M* β-mercapto­ethanol at a flow rate of 0.5 ml min^−1^.

### 2.4. Small-angle X-ray scattering (SAXS) experiments   

SAXS data were obtained at the European Synchrotron Radiation Facility (ESRF), Grenoble, France. The data were recorded on beamline BM-29 and absolute-scale calibration was performed with bovine serum albumin (BSA) and water as references. The obtained data were azimuthally averaged, normalized and background-subtracted using the *BsxCuBE* software suite available at the beamline (Pernot *et al.*, 2010 ▶). This yielded the scattering intensity *I*(*q*), where the scattering vector *q* is defined by *q* = 4πsin(θ)/λ, where θ is half of the scattering angle and λ is the wavelength of the incoming beam. All modelling was performed with *CORAL* (Petoukhov *et al.*, 2012 ▶) and the scattering from all structures was evaluated with *CRYSOL* (Svergun *et al.*, 1995 ▶). *CORAL* runs were made without any imposed symmetry and *CRYSOL* was run using the default settings. Both software packages are from the *ATSAS* suite v.2.4 (Petoukhov *et al.*, 2012 ▶).

### 2.5. Microscale thermophoresis binding studies   

Protein interactions with chitohexaose were assessed using microscale thermophoresis (MST; Seidel *et al.*, 2013 ▶). Proteins were labelled using the Monolith NT.115 Protein Labeling Kit BLUE (NanoTemper Technologies), and a labelling efficiency of approximately 2:1 molar ratio of labelled protein to dye was achieved. A twofold titration series was prepared in which the concentration of the labelled proteins was kept constant at 200 n*M* and the concentration of the titrant, chitohexaose, was varied from 152 n*M* to 5 m*M* in thermophoresis buffer consisting of 50 m*M* phosphate pH 7.5 and 0.1% Tween 20. After incubation for 1 h at 60°C in the dark, MST measurements were performed at room temperature on a Monolith NT.115 instrument (NanoTemper Technologies). Standard capillaries were used and the LED power was adjusted to 50%. Negative controls for each protein were performed using 200 n*M* labelled protein in thermophoresis buffer in all 16 capillaries under the same conditions as mentioned above. For each measurement the laser was switched on for 30 s and off for 5 s. Binding curves were obtained from the thermophoresis phase with an infrared laser power of 20%. For each protein, three sets of titration series were prepared and the sigmoidal dose-response curves were fitted with *GraphPad Prism* 6 to yield an average *K*_d_ value.

### 2.6. PDB codes   

The atomic coordinates and structure factors of the structures of P60_tth, P60_2LysM bound to chitohexaose and P60_2LysM have been deposited in the Protein Data Bank (Berman *et al.*, 2000 ▶) as entries 4xcm, 4uz3 and 4uz2, respectively.

## 3. Results and discussion   

### 3.1. Crystal structure of P60_tth   

The P60_tth protein was expressed and crystallized as described previously (Wong & Blaise, 2013 ▶). The P60_tth structure was solved by the single-wavelength anomalous dispersion (SAD) method (Hendrickson & Teeter, 1981 ▶) using selenomethionine-derivative protein as described previously (Wong & Blaise, 2013 ▶).

The structure was refined to 2.65 Å resolution and the refinement statistics are shown in Table 1 ▶. The final model contained two molecules in the asymmetric unit (chains *A* and *B*). The molecules are not equivalent in terms of model-building completion. In the two catalytic domains, residues 117–246 could be built for chain *A* and residues 122–245 for chain *B*. Only three of the four LysM domains could be traced; the two N-terminal LysM domains (LysM1) could be modelled but no electron density was observed for one of the second LysM domains: LysM2 from chain *A*. In addition, the linker between LysM1 and LysM2 of chain *B* could be traced (Figs. 1 ▶ *b* and 1 ▶ *c*).

**Figure 2:**
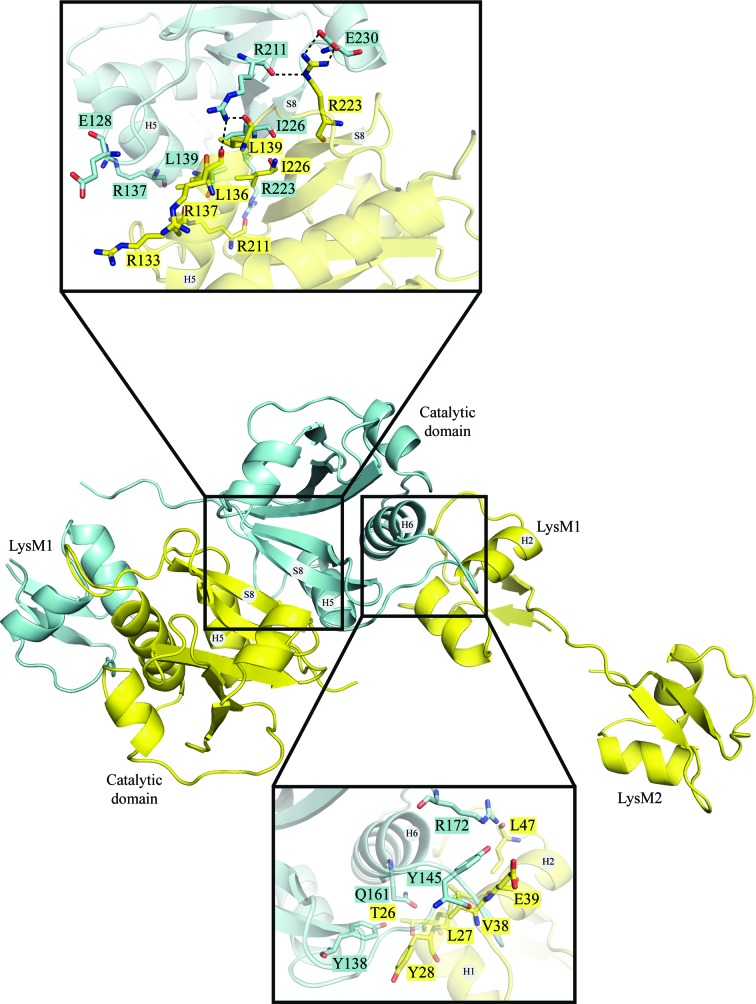
Dimerization interface of P60_tth. The central figure shows the overall dimerization through the catalytic domains and interaction between the LysM1 and catalytic domains. The upper panel displays an enlarged view of the dimerization interface between the two catalytic domains as seen from the back of the central figure. All residues involved in the dimerization interface formed by hydrogen bonds, salt bridges or hydrophobic interactions are represented by sticks and are labelled with single-letter amino-acid codes. The lower panel displays an enlarged view of the interaction interface between the LysM1 and catalytic domains.

Analysis of the crystal packing with the *PISA* server (Krissinel & Henrick, 2007 ▶) indicates that a stable homodimer is formed within the crystal. Dimer formation is mediated by interactions between the two catalytic domains and between the LysM1 domain of chain *B* and the catalytic domain of chain *A* and *vice versa* (Figs. 1 ▶ *b* and 2 ▶). The catalytic domains dimerize *via* a surface area of about 980 Å^2^. This dimerization interface involves 17 residues mainly belonging to strand 8 (S8) and helix 5 (H5) of each monomer (Fig. 2 ▶). Two salt bridges are established between the side chains of Arg223 and Glu230 from each catalytic domain. Additionally, 11 hydrogen bonds and van der Waals interactions stabilize the dimer interface (Fig. 2 ▶, upper panel). The interaction surface between LysM1 and the catalytic domain is 593 Å^2^. This interface involves residues in the vicinity of H6 from the catalytic domain and residues from H1 and H2 of LysM1. 21 residues of the catalytic domain contact 14 residues of the LysM1 domain. Six of these interactions are mediated by hydrogen bonds (Fig. 2 ▶, lower panel). We analyzed all of the NlpC/P60 structures deposited in the PDB and observed that three NlpC/P60 proteins with the PDB codes 4hpe (Joint Center for Structural Genomics, unpublished work), 3pvq (Joint Center for Structural Genomics, unpublished work) and 2evr (Xu *et al.*, 2009 ▶) seem to be able to form stable homodimers. Nevertheless, none of them have the same dimer interface as observed in the P60_tth structure (not shown).

The structure can be divided into two parts: the anchoring domain at the N-terminus and the catalytic domain at the C-terminus (Fig. 1 ▶ *a*). The anchoring domain is made up of two LysM domains that are connected to the catalytic domain by a polyproline linker that could not be traced (Fig. 1 ▶ *c*).

The catalytic domain is made up of a central β-sheet composed of five antiparallel β-strands that are surrounded by four α-helices (Figs. 1 ▶ *c* and 3 ▶ *a*). A search for structurally related proteins using the *DALI* server (Holm & Rosenström, 2010 ▶) shows that the catalytic domain matches structures from the NlpC/P60 protein family. The most similar structures are the putative cell-wall hydrolase from *Clostridium difficile* (PDB entry 4hpe; Joint Center for Structural Genomics, unpublished work), with a *Z*-score of 16.3 and a root-mean-square deviation (r.m.s.d.) of 2.7 Å over the C^α^ atoms of 116 residues, and the d,l-endopeptidase YkfC from *B. cereus* (PDB entry 3h41; Xu *et al.*, 2010 ▶), with a *Z*-score of 16.1 and an r.m.s.d. of 1.9 Å over the C^α ^ atoms of 111 residues. These two catalytic domains share 32% sequence identity with the catalytic domain of P60_tth. The comparison to the YkfC structure is of interest since it was solved with a bound ligand: the l-Ala-d-Glu peptide (Xu *et al.*, 2010 ▶). As such, we can use the YkfC model to identify the putative catalytic residues of P60_tth and to propose its probable function.

**Figure 3:**
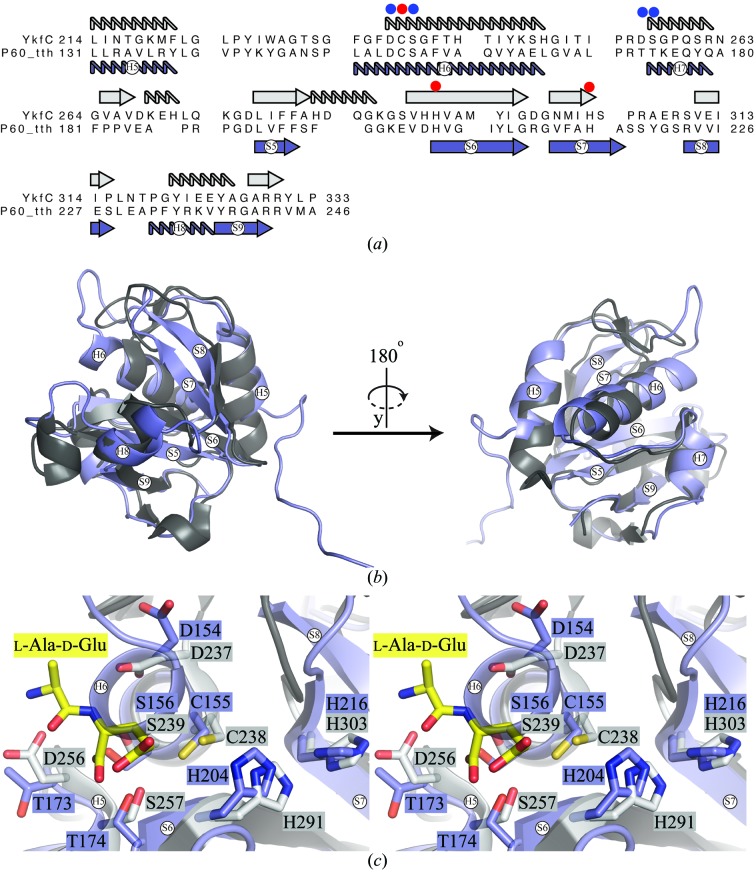
Structure comparison of the catalytic domain of P60_tth with YkfC from *B. cereus*. (*a*) Primary-sequence alignment of P60_tth with YkfC from *B. cereus*. The secondary structures of the two proteins are indicated. The red sphere indicates the catalytic triad, while the blue spheres indicate other residues that are predicted to be involved in forming the catalytic site. (*b*) Superposition of the three-dimensional crystal structures of P60_tth (violet) and *B. cereus* YkfC (grey; PDB entry 3h41). The strand and helix numbering corresponds to that of P60_tth. (*c*) Comparison of active sites in the two crystal structures displayed as a cross-eyed stereoview. The colour code is the same as in (*b*).

Superposition of the P60_tth and YkfC structures indicates that the two catalytic domains are indeed highly similar (Figs. 3 ▶ *b* and 3 ▶ *c*). The Cys, His and His catalytic triad in the active site of YkfC is conserved in P60_tth (Fig. 3 ▶ *c*). Moreover, all residues whose side chains are involved in the binding of the l-Ala-d-Glu product are either semi-conserved or fully conserved (Fig. 3 ▶ *c*). We notice, however, that H6 of the catalytic domain is one turn longer in P60_tth compared with YkfC. Consequently, a steric clash is observed between H6 of P60_tth and the l-Ala-d-Glu product in YkfC when superposing the two structures (Fig. 3 ▶ *c*). This indicates that the substrate/product of P60_tth may differ from that of YkfC and/or that the catalytic site requires some structural rearrangement before it can bind its substrate/product. Overall, comparisons to known NlpC/P60 structures strongly suggest that P60_tth also functions as a d,l-endopeptidase involved in PGN hydrolysis.

Despite numerous efforts, we have not been able to identify any hydrolytic activity of the P60_tth protein on *E. coli*, *B. subtilis* or *T. thermophilus* cells or purified cell walls. We have also tried unsuccessfully to assess the *in vitro* activity of P60_tth on commercial PGN fragments and chemically synthesized cross-linked PGN peptides from *T. thermophilus* (Supporting Information). This absence of activity is puzzling, but similar difficulties in establishing NlpC/P60 enzymatic assays have also been reported recently (Gomez *et al.*, 2014 ▶). Furthermore, it cannot be excluded that we did not identify the optimal conditions for P60_tth activity and/or that the enzyme needs to undergo proteolytic activation, as demonstrated for the *M. tuberculosis* NlpC/P60 protein RipA (Ruggiero *et al.*, 2010 ▶; Chao *et al.*, 2013 ▶).

The anchoring domain is composed of two LysM domains (Fig. 1 ▶ *a*). Each LysM domain adopts a βααβ fold (Figs. 1* ▶c* and Supplementary Fig. S1). The primary sequences of LysM1 and LysM2 are very similar since they share 72% sequence identity. Superposition of the LysM domains yields an r.m.s.d. of 0.55 Å over the main chain of 42 residues.

The two LysM domains are very similar to the LysM structures deposited in the PDB, notably to the LysM domain of the *B. subtilis* YkuD protein (Bielnicki *et al.*, 2006 ▶; Lecoq *et al.*, 2012 ▶), the LysM2 domain of the fungal Ecp6 protein (Sánchez-Vallet *et al.*, 2013 ▶) and the LysM2 domain of the plant CERK1 receptor (AtCERK1; Liu *et al.*, 2012 ▶) (Supplementary Fig. S1). The main difference is the existence of an extra helix turn between H2 and S2 in both the AtCERK1 and the YkuD LysM structures; only a loop is present in the corresponding region of P60_tth LysM1 (Supplementary Fig. S1).

### 3.2. P60_tth is a homodimer in solution   

**Figure 4:**
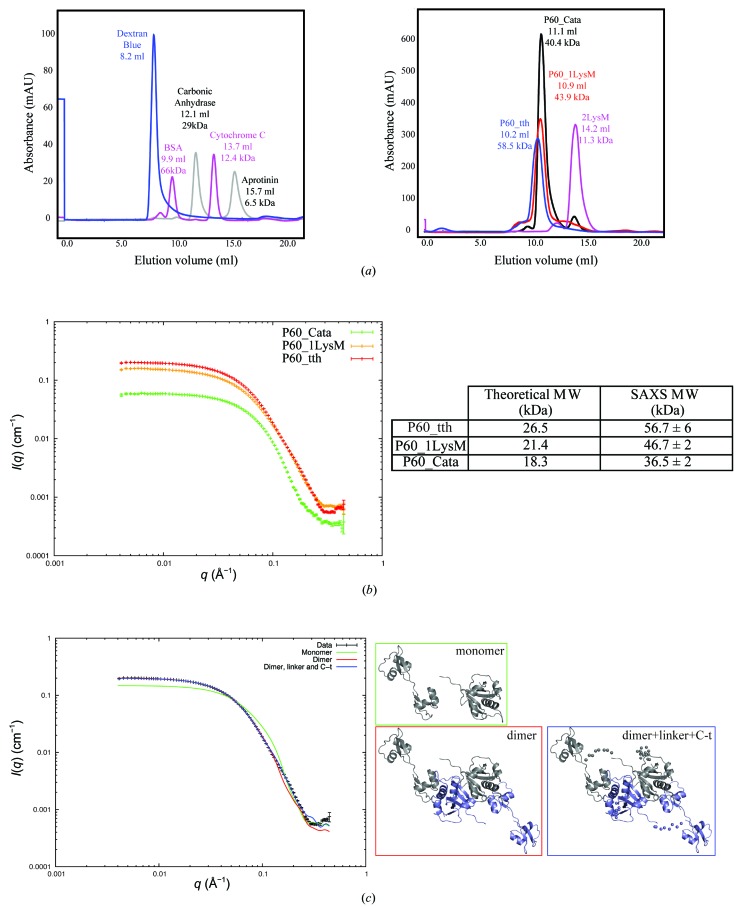
Determination of the oligomeric state and structure of P60_tth in solution. (*a*) Size-exclusion chromatography of P60_tth and truncated mutants on a Superdex 75 column. The left panel shows the calibration curve and the right panel shows the elution profile of the different proteins. The elution volume and calculated apparent molecular weight are indicated above each peak. P60_tth is the full-length protein; in P60_cata, only the catalytic domain is present. In P60_1LysM, the N-terminal LysM domain has been deleted and in P60_2LysM the catalytic domain has been truncated. (*b*) Calculated molecular weight derived from SAXS data. The left panel shows the SAXS data and the table on the right compares the theoretical molecular weight calculated from the primary sequence with the apparent molecular weight established from the SAXS data. (*c*) Modelling of P60_tth in solution. The left plot represents the *CRYSOL* evaluation of the three models shown in the right panel. The plots clearly show that the model of the dimer fits the SAXS data better than the model of the monomer and that *CORAL* modelling of the linkers into the dimeric model further improves the fit.

To investigate whether the homodimer exists in solution, we first estimated the oligomeric state of P60_tth using size-exclusion chromatography (SEC). The chromatogram indicates that the full-length protein (P60_tth) has an apparent molecular weight of 58.5 kDa (Fig. 4 ▶ *a*). This corresponds to a dimer since the theoretical molecular weight of the monomer is 26.5 kDa. From the crystal-packing analysis, it seems that the strongest interactions are established between the two catalytic domains. To verify this, we expressed and purified truncated versions of the protein in which one (P60_1LysM) or two (P60_cata) LysM domains were deleted. These two proteins with predicted molecular weights of 21.4 and 18.3 kDa, respectively, eluted with apparent molecular weights of 43.9 and 40.4 kDa, respectively, which corresponds to dimers (Fig. 4 ▶ *a*). In contrast, a construct possessing only the two LysM domains (P60_2LysM), *i.e.* without a catalytic domain, with a predicted molecular weight of 10.9 kDa, elutes with an apparent mass of 11.2 kDa, reflecting the presence of a monomer (Fig. 4 ▶ *a*). In summary, the SEC experiments indicate that P60_tth is a homodimer in solution and that the catalytic domains mediate the dimerization.

Additionally, small-angle X-ray scattering (SAXS) data were collected for the full-length and LysM-truncated forms of the protein (Fig. 4 ▶ *b*). The estimated molecular weight of the full-length protein is 56.7 kDa, supporting our observation that P60_tth forms stable dimers in solution. The P60_1LysM and P60_cata truncation mutants also behave as dimers in solution. Hence, the SAXS experiments confirm the existence of stable dimers in solution.

Furthermore, we combined our SAXS and crystallographic data to model the complete P60_tth dimer. To do so, we superposed the most complete molecule from the crystal structure with the less complete molecule, *i.e.* chain *B* was superposed on chain *A*. This dimeric model (dimer), a monomeric model (monomer) and a dimeric model including dummy residues representing the amino acids not seen in the crystal structure (dimer + linker + C-t) were evaluated against the SAXS data (Fig. 4 ▶ *c*). *CORAL* (Petoukhov *et al.*, 2012 ▶) was used to model the missing residues and the evaluation of the fit of the models to the SAXS data was performed with *CRYSOL* (Svergun *et al.*, 1995 ▶). The resulting fits (Fig. 4 ▶ *c*) showed a clear improvement from the monomer (χ value of 97.34) to the dimer (χ value of 10.97). The fit was further improved when the dimeric model containing dummy residues (χ value of 7.29) was used in the evaluation (Fig. 4 ▶ *c*). Further refinement of the model did not improve the fit significantly, thereby confirming that the solution structure is highly similar to the crystal structure.

### 3.3. LysM domains cooperate to bind long carbohydrates   

**Figure 5:**
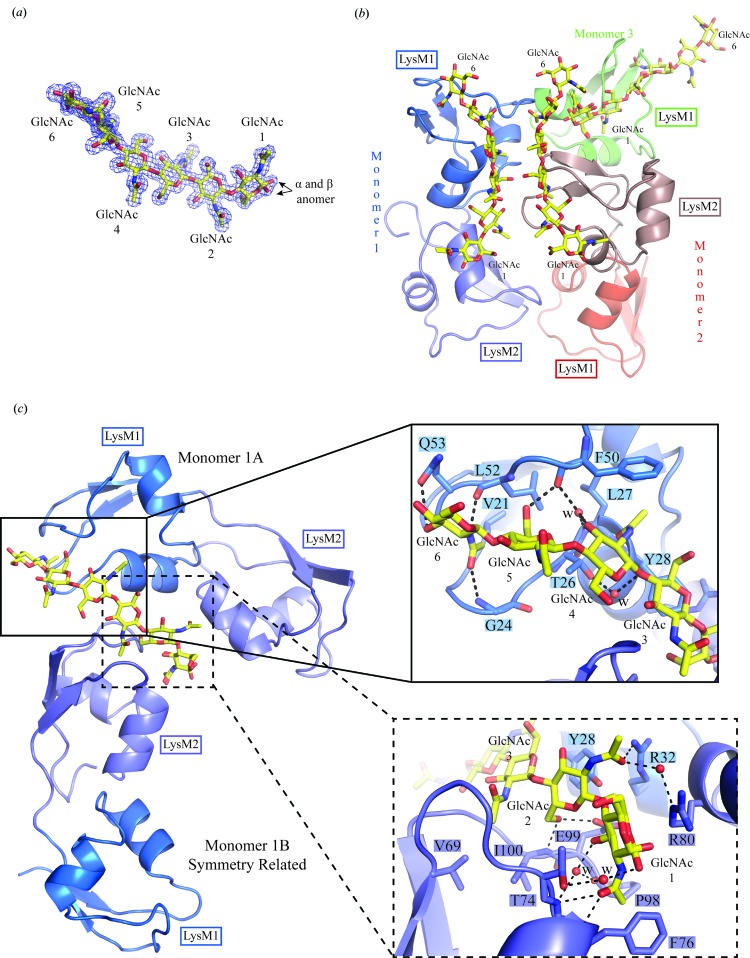
Crystal structure of P60_2LysM bound to chitohexaose. (*a*) 2*F*_o_ − *F*_c_ OMIT map. The map contoured at the 1σ level was calculated with *phenix.autobuild* from the *PHENIX* package after omitting the chitohexaose molecules present in the asymmetric unit. The arrows indicate the α and β anomers, the occupancies of which were calculated to be 0.5. (*b*) Composition of the asymmetric unit. The asymmetric unit is composed of five LysM domains and three molecules of chitohexaose. (*c*) Recognition of chitohexaose. The left panel shows how chitohexaose is recognized by symmetry-related molecules. The two right panels are an enlarged view of the recognition of GlcNAc 6 to GlcNAc 3 (upper panel) and GlcNAc 3 to GlcNAc 1 (lower panel). Residues represented in marine blue or slate blue are from LysM1 and LysM2, respectively. The dashed lines indicate hydrogen bonds.

Since we aimed to understand how LysM domains anchor the catalytic domain onto PGN, we tried to obtain a co-crystal structure of full-length P60_tth bound to ligands, unfortunately without any success. Attempts to soak chitin and PGN carbohydrate polymers into P60_tth crystals were also futile. Alternatively, we tried to co-crystallize the construct containing only two LysM domains, P60_2LysM (Fig. 1 ▶ *a*). As PGN fragments with long MurNAc-GlcNAc chains are very difficult to obtain, we tried to co-crystallize P60_2LysM with GlcNAc polymers. This approach is relevant since we have shown previously that bacterial LysM domains bind MurNAc-GlcNAc and GlcNAc polymers with similar affinities (Wong *et al.*, 2014 ▶). Using this strategy, we successfully crystallized and solved the crystal structure of P60_2LysM bound to chito­hexaose (Figs. 5 ▶ *a* and 5 ▶ *b*).

The structure was solved to 1.75 Å resolution (Table 1 ▶). Three molecules of P60_2LysM (monomers 1–3) are present in the asymmetric unit (Fig. 5 ▶ *b*). Monomers 1 and 2 are identical, while no electron density was observed for the LysM2 domain of monomer 3. All LysM domains that could be traced, however, bind a chitohexaose molecule (Fig. 5 ▶ *b*).

The LysM binding cleft is similar to those described for the crystal structures of the plant CERK1 receptor (Liu *et al.*, 2012 ▶) and the fungal Ecp6 protein (Sánchez-Vallet *et al.*, 2013 ▶) and the NMR solution structures of the bacterial AtlA autolysin (Mesnage *et al.*, 2014 ▶) and the fungal CVNH-LysM lectin (Koharudin *et al.*, 2011 ▶). A similar LysM binding cleft has also been characterized biochemically by NMR for plant chitinase A (Ohnuma *et al.*, 2008 ▶). The binding pocket is delimited by the loop between S1 and H1 and the loop between H2 and S2. Monomers 1 and 2 bind chitohexaose in the same manner, but differently from monomer 3. For monomers 1 and 2, the carbohydrate induces intermolecular dimerization with symmetry-related molecules (Fig. 5 ▶ *c*).

LysM1 of monomer 1*A* (Fig. 5 ▶ *c*) mainly contacts GlcNAc 6 to GlcNAc 3. The side chain of Gln53 contacts O3 of GlcNAc 6, while the main chains of Gly24 and Leu52 contact its *N*-acetyl group. Additionally, the side chain of Val21 mediates a hydrophobic interaction with the *N*-acetyl group of GlcNAc 6. The main chain of Phe50 recognizes O6 of GlcNAc 5. Phe50 also mediates a hydrogen bond to GlcNAc 4 *via* a water molecule that is stabilized by the main chain of Leu27. Furthermore, the Phe50 side chain mediates a hydrophobic interaction with the *N*-acetyl group of GlcNAc 4, which is also recognized by the main chain of Tyr28. Finally, Thr26 mediates a hydrogen bond *via* O4 of GlcNAc 3.

The recognition of GlcNAc 3, GlcNAc 2 and GlcNAc 1 is achieved by LysM1 and LysM2 of monomer 1*A* and LysM2 of the symmetry-related monomer 1*B*. The side chain of Val69 mediates a hydrophobic interaction with the *N*-acetyl group of GlcNAc 3, while the main chains of Ile100 and the carboxylic group of Glu99 establish hydrogen bonds to O6 of GlcNAc 2. The main chains of Pro98 and Leu75 stabilize a water molecule which mediates a hydrogen bond to O3 of GlcNAc 1, while the main chains of Leu75 and Phe76 recognize O7 of the *N*-acetyl group. Thr74 binds the O1 group of GlcNAc 1. Finally, Arg32 and Arg80 from LysM1 and LysM2 of monomer 1*A*, respectively, recognize GlcNAc 2 *via* two water-mediated hydrogen bonds.

**Figure 6:**
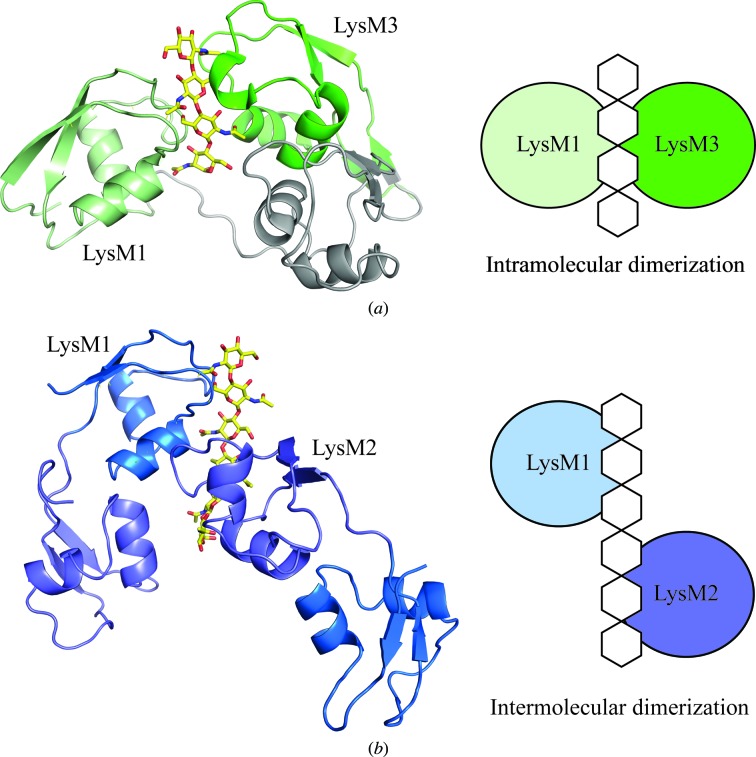
Comparison of the dimerization mode between bacterial and fungal LysM domains. (*a*) Intramolecular dimerization mode of chitin binding observed in the fungal Ecp6 protein (PDB entry 4b8v; Sánchez-Vallet *et al.*, 2013 ▶). LysM1 and LysM3 involved in carbohydrate binding are coloured light and dark green, respectively. (*b*) Intermolecular dimerization mode of chitin binding observed in the bacterial P60_2LysM protein; the colour code is the same as in Fig. 5 ▶.

In summary, we observed that LysM1 of monomer 1*A* mainly contacts the last four GlcNAc residues (GlcNAc 6 to GlcNAc 3), while LysM2 of monomer 1*B* mainly contacts the first three GlcNAc residues (GlcNAc 3 to GlcNAc 1); this LysM2 domain could also potentially interact with a fourth GlcNAc residue if a longer chitin polymer was present. There is a strong difference between this intermolecular dimerization mode and the intramolecular dimerization mode observed in the crystal structure of fungal Ecp6 bound to chitin (Fig. 6 ▶; Sánchez-Vallet *et al.*, 2013 ▶). In the Ecp6 structure, the four GlcNAc residues are sandwiched between the two intrachain LysM domains. This sandwich mode of binding has also recently been proposed to occur for chitin recognition by the CERK1–OsCEBiP complex involved in rice immune responses (Hayafune *et al.*, 2014 ▶). Our structure offers an alternative binding mode that could explain how LysM receptors dimerize and signal upon recognition of long chitin oligomers.

Very interestingly, the fact that chitohexaose is recognized by two LysM domains from different monomers supports several biochemical observations made on LysM proteins from different phyla (Wong *et al.*, 2014 ▶; Hayafune *et al.*, 2014 ▶; Liu *et al.*, 2012 ▶). We and others have recently proposed that LysM domains in multiple LysM-containing proteins act cooperatively to enhance the binding of these proteins to long carbohydrates (Wong *et al.*, 2014 ▶; Mesnage *et al.*, 2014 ▶). However, we could not explain whether this was owing to the fact that each LysM domain can bind a chitin molecule or because several LysM domains can bind to the same chitin molecule. With our crystal structure, we now claim that both events occur, since each LysM domain in the asymmetric unit binds a chito­hexaose molecule which could also be bound by a LysM domain from a different monomer.

Dimerization of LysM domains through carbohydrates has also been demonstrated to be very important for LysM receptors involved in plant defence and symbiotic mechanisms (Hayafune *et al.*, 2014 ▶; Liu *et al.*, 2012 ▶; Madsen *et al.*, 2011 ▶), and models of dimerization have been proposed. Our crystal structure now provides the structural basis for these observations and therefore aids in the design of receptor-dimerization models that are of great importance in this field of research.

### 3.4. Comparison of the chitin-binding site in LysM from different phyla   

**Figure 7:**
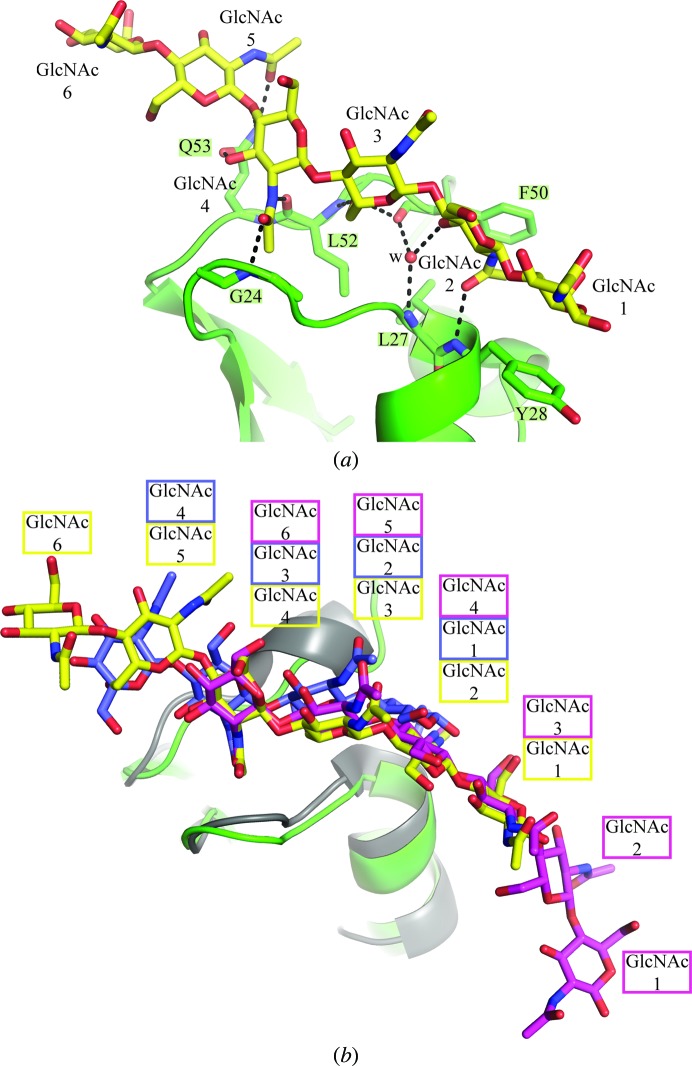
Second binding mode observed in the asymmetric unit. (*a*) Interaction of chitohexaose as seen in monomer 3 in the asymmetric unit. (*b*) Comparison of the chitin-binding sites in the LysM domains of P60_2LysM and AtCERK1. Superposition of the chitin molecules as observed in monomers 1 and 3 of our P60_2LysM–chitohexaose crystal structure (green) and the LysM2 domain (grey) of the AtCERK1–chitopentaose crystal structure. Chitohexaose molecules from monomers 1 and 3 are displayed in pink and yellow, respectively, while the chitin molecule from AtCERK1 is displayed in violet.

Although a chitohexaose molecule occupies the same binding pocket in the LysM1 domain of monomer 3, a second type of binding is observed (Fig. 7 ▶ *a*). This LysM1 domain recognizes GlcNAc 5 to GlcNAc 1, while GlcNAc 6 is not contacted. The Gln53 side chain binds to both GlcNAc 5 and GlcNAc 4, and the main chains of Gly24 and Leu52 bind to the *N*-acetyl group of GlcNAc 4. The main chains of Phe50 and Leu52 mediate interactions with the O6 group of GlcNAc 3, while the main chains of Leu27 and Phe50 bind to O3 of GlcNAc 2. Finally, the main chain of Tyr28 contacts the *N*-acetyl group of GlcNAc 2, while the side chain stacks with GlcNAc 1.

By comparing the two different positions of the chitohexaose molecules observed in the LysM1 domains (monomers 1*A* and 3) of P60_tth with the position of the chitopentaose molecule observed in the LysM2 domain of the plant AtCERK1 receptor crystal structure (Liu *et al.*, 2012 ▶), we see that the GlcNAc 6 position in the LysM binding site of monomer 1*A* (Fig. 6 ▶) corresponds to the GlcNAc 4 position in monomer 3 and the GlcNAc 3 position in AtCERK1 LysM2 (Figs. 5 ▶ *c* and 7 ▶ *a*). It is therefore tempting to propose that the LysM domains might be able to ‘slide’ along carbohydrates.

### 3.5. Mutations in the LysM binding site affect chitohexaose dissociation constants   

To further validate that the binding site observed in the crystal structure is biologically relevant, we used an alanine-scanning approach to mutate residues in the binding site. Subsequently, microscale thermophoresis (MST; Seidel *et al.*, 2013 ▶) was used to measure binding affinities towards chito­hexaose in solution, as it has previously been shown to be suitable for measuring such interactions (Wong *et al.*, 2014 ▶; Maolanon *et al.*, 2014 ▶; Broghammer *et al.*, 2012 ▶).

We compared the binding capacity of full-length P60_tth to two mutant proteins, P60_tth_LysM1_mut (Y28A, R32A, F50A, Q53A) and P60_tth_LysM2_mut (F76A, R80A, E99A), in which the residues involved in carbohydrate binding *via* side-chain interactions were mutated to Ala (Supplementary Fig. S2). The full-length P60_tth protein has an apparent *K*_d_ of 90 ± 19.8 µ*M* for chitohexaose (Supplementary Fig. S2). P60_tth_LysM1_mut and P60_tth_LysM2_mut have similar *K*_d_ values of 320.7 ± 112.3 and 292.1 ± 86.5 µ*M*, respectively, which are approximately three times lower than that of the wild-type protein (Supplementary Fig. S2). It appears that numerous interactions between the carbohydrate and the protein main chain may be sufficient to preserve binding. Although the mutations did not abolish protein–carbohydrate interactions, the reduced binding affinities help to validate the biological relevance of the chitohexaose binding site observed in the crystal structure. Considering the similarity of the binding sites determined in this bacterial endopeptidase LysM domains to the binding sites observed in plant and fungal LysM domains (Sánchez-Vallet *et al.*, 2013 ▶; Liu *et al.*, 2012 ▶; Ohnuma *et al.*, 2008 ▶; Koharudin *et al.*, 2011 ▶), we conclude that LysM–carbohydrate binding sites are conserved among phyla.

### 3.6. Multiple LysM domains are flexible   

We have also successfully solved the crystal structure of P60_2LysM without any ligand (Table 1 ▶). By comparing the LysM domains in this structure with the LysM domains in the full-length P60_tth and the P60_2LysM–chitohexaose structures, we observe that binding of the carbohydrate triggers only minor structural rearrangements in the binding pocket. Only the side chains of Gln53 and Arg32 reorient upon carbohydrate binding (not shown).

**Figure 8:**
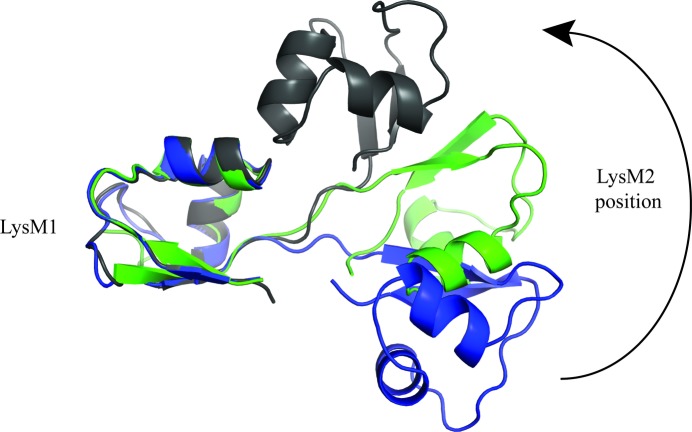
Comparison of the relative positions of the LysM2 domains in the three crystal structures. The figure shows that the linker between the two LysM domains confers flexibility between LysM domains. The positions of the LysM2 domains from full-length P60_tth (grey), P60_2LysM bound to chitohexaose (green) and P60_2LysM free from ligand (blue) are compared.

However, the relative positions of the two LysM domains differ significantly in the bound state compared with the unbound states (Fig. 8 ▶). The linker seems to allow some flexibility between LysM domains, but we cannot claim for certain that the movement is triggered by carbohydrate binding because such movements may arise owing to crystal packing. Nonetheless, we recently showed through SAXS experiments that the four LysM domains of the *B. subtilis* CwlS protein are flexible in solution (Wong *et al.*, 2014 ▶). Our structural data reinforce this observation and clearly indicate that the two LysM domains in P60_tth are flexible despite being separated by a short linker of only four amino acids.

### 3.7. A model of P60_tth interacting with peptidoglycan   

Although we could not obtain a crystal structure of P60_tth bound to PGN fragments, the structures of the full-length P60_tth and chitohexaose-bound P60_2LysM enable us to propose a model suggesting how NlpC/P60 proteins possessing multiple LysM domains might recognize PGN.

**Figure 9:**
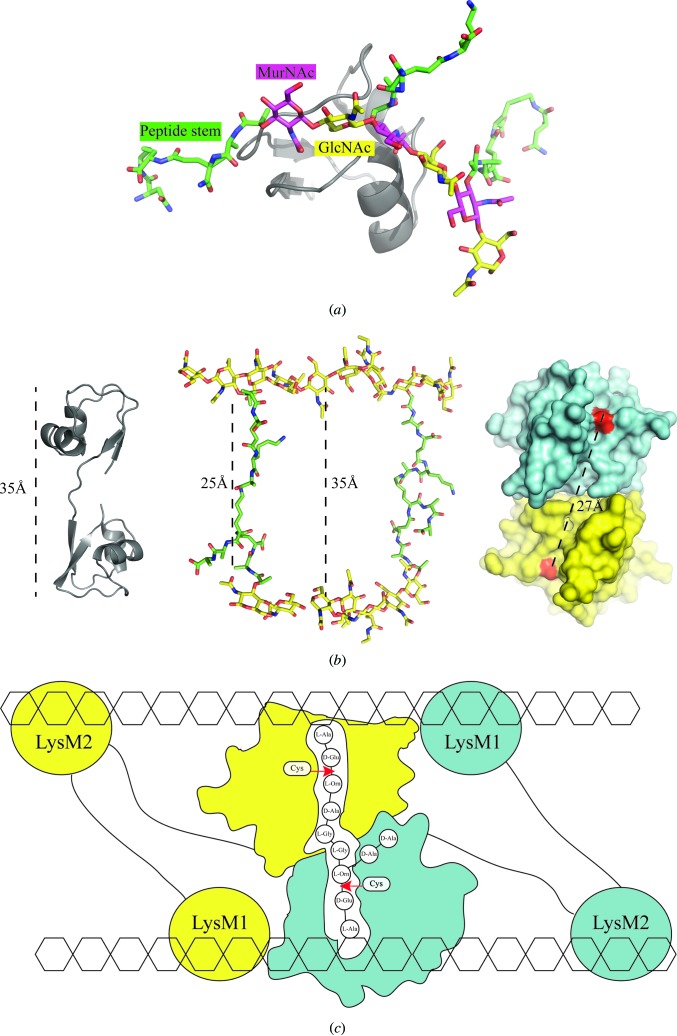
Proposed model of PGN recognition by P60_tth. (*a*) PGN recognition model depicting how the LysM domain interacts with a PGN fragment. Each MurNAc is linked to a peptide [l-Ala-γ-d-Gln-l-Lys-(d-Asn)]; the MurNAc-peptide molecule was extracted from the l,d-carboxypeptidase crystal structure (PDB entry 4oxd; Hoyland *et al.*, 2014 ▶). (*b*) Distances observed between cross-linked PGN strands and the length of the peptide stem in the three-dimensional model of *S. aureus* PGN proposed by Meroueh *et al.* (2006 ▶). The distance between the two LysM binding sites in the P60_tth full-length crystal structure and between the entrance of the two active sites (red) of the P60_tth catalytic domains are also indicated. (*c*) Scheme explaining how the P60_tth homodimer could anchor the protein onto PGN. The PGN GlcNAc-MurNAc strands are represented by hexagons and the cross-linked peptide-stem composition of *T. thermophilus* is indicated by three-letter amino-acid codes; the amino-acid composition has been described previously (Quintela *et al.*, 1995 ▶). ‘Cys’ represents the catalytic cysteines and the red arrows indicate the putative cleavage sites in the peptide stem.

First of all, we superposed a MurNAc peptide (Hoyland *et al.*, 2014 ▶) onto GlcNAc 6, GlcNAc 4 and GlcNAc 2 of the chitohexaose from our P60_2LysM–chito­hexaose structure. With minimal additional modelling (rotating only the bond between l-Ala and MurNAc), we could fit the peptide stem without inducing any steric hindrance with the residues from the LysM binding site (Fig. 9 ▶ *a*). This suggests that a MurNAc-GlcNAc oligosaccharide might interact in a similar way to that observed with a GlcNAc oligosaccharide and that the peptide portion of PGN might not be recognized at all by the residues in the LysM domains. However, we do not exclude the possibility that the peptide portion of PGN might trigger steric hindrance upon binding in the LysM groove. This hypothesis was demonstrated in a recent study by Mesnage and coworkers, who proposed that the peptide portion of PGN reduces the affinity of the *Enterococcus faecalis* AtlA single LysM domain for PGN (Mesnage *et al.*, 2014 ▶).

The distance between the binding sites of the two LysM domains is about 35 Å, which interestingly is the same as the distance between MurNAc-GlcNAc strands that are cross-linked by the PGN peptide stem in the three-dimensional model of PGN proposed by Meroueh *et al.* (2006 ▶). Moreover, the distance between the two putative catalytic cysteines of the catalytic domain is about 27 Å, while the length of the peptide stem is about 25 Å in the PGN model (Fig. 9 ▶ *b*). These simple distance observations led us to propose a model of interaction in which individual LysM domains bind opposite carbohydrate strands of PGN, enabling favourable positioning of the catalytic domains to cleave the peptide stems (Fig. 9 ▶ *c*). With the knowledge that P60_tth behaves as a homodimer, and assuming that the protein cleaves the peptide arm between the second and third amino acids, we postulate that the two catalytic domains might cleave two bonds simultaneously. This could confer an advantage since PGN fragments released during PGN remodelling are recycled (Reith & Mayer, 2011 ▶; Boudreau *et al.*, 2012 ▶; Johnson *et al.*, 2013 ▶). The P60_tth homodimer could release two GlcNac-MurNac peptides in each hydrolysis step instead of one, therefore enhancing the PGN recycling efficiency.

## 4. Concluding remarks   

In this study, we present a novel structure of an NlpC/P60 protein containing multiple LysM domains. Additionally, the structure of P60_2LysM bound to chitohexaose provides the first structural evidence for intermolecular dimerization of LysM-containing proteins on a GlcNAc polymer. Based on investigations of the crystal structures of P60_tth and P60_2LysM, we have proposed models describing how bacterial LysM domains recognize PGN and how the dimerization of the LysM and catalytic domains may be features that enhance the recognition of PGN and efficiency of PGN hydrolysis by P60_tth.

## 5. Related literature   

The following references are cited in the Supporting Information for this article: Agnihotri *et al.* (2011 ▶), Kok *et al.* (2009 ▶) and Kumar *et al.* (2013 ▶).

## Supplementary Material

PDB reference: P60_tth, 4xcm

PDB reference: P60_2LysM, 4uz2

PDB reference: P60_2LysM bound to chitohexaose, 4uz3

Supporting Information.. DOI: 10.1107/S139900471402793X/rr5089sup1.pdf

## Figures and Tables

**Table 1:** Data collection and refinement statistics Values in parentheses are for the last resolution shell.

	Full-length P60_tth, Se peak	P60_2LysMchitohexaose	P60_2LysM
Data-collection statistics			
Beamline	I911-3, MAX-lab	I911-2, MAX-lab	I911-3, MAX-lab
Wavelength ()	0.978	1.041	0.976
Space group	*P*6_1_	*P*2_1_3	*P*4_2_2_1_2
Unit-cell parameters			
*a* = *b* ()	71.6	105.4	122.9
*c* ()	197.8	105.4	76.8
Resolution ()	302.60 (2.652.60)	201.75 (1.801.75)	202.50 (2.602.50)
*R*_meas_ (%)	6.9 (75.7)	12 (87.6)	8.2 (81.6)
*I*/(*I*)	15.3 (2.1)	20.4 (3.3)	24.1 (3.2)
Completeness (%)	99.8 (99.8)	99.9 (100)	98.8 (99.8)
Multiplicity	5.9 (5.9)	12.3 (12.3)	11.7 (11.9)
Refinement statistics			
Resolution ()	29.52.65	201.75	202.50
No. of reflections	16618	39631	20657
*R*_work_/*R*_free_ (%)	19.4/23.8	15.1/18.9	21.2/25.5
No. of atoms			
Protein	3056	1971	3047
Water	16	393	82
Ligand		327	
Average *B* values (^2^)			
Protein, overall	96.1	18.7	71.7
Water	16	32.3	49.7
Ligand		17	
R.m.s. deviations			
Bond lengths ()	0.003	0.005	0.002
Bond angles ()	0.71	0.92	0.55
PDB code	4xcm	4uz3	4uz2
