# Supplementary material for: An intermolecular binding mechanism involving multiple LysM domains mediates carbohydrate recognition by an endopeptidase
Source: Acta Crystallogr D Biol Crystallogr. 2015 Feb 26;71(Pt 3):592–605. doi: 10.1107/S139900471402793X (PMC4356369; doi:10.1107/S139900471402793X)
Supplement: Supplementary file 1 [file d-71-00592-sup1.pdf]

BIOLOGICAL
CRYSTALLOGRAPHY

Volume 71 (2015)

Supporting information for article:

An intermolecular binding mechanism involving multiple LysM domains mediates carbohydrate recognition by an endopeptidase

Jaslyn E. M. M. Wong, Søren Roi Midtgaard, Kira Gysel, Mikkel B. Thygesen, Kasper K. Sørensen, Knud J. Jensen, Jens Stougaard, Søren Thirup and Mickaël Blaise

S1. Synthesis of *T. thermophilus* peptidoglycan fragment and activity assay

S1.1. Synthesis of peptidoglycan fragments:

The peptides were prepared by Fmoc solid-phase peptide synthesis on a Biotage® Initiator+ Alstra™ microwave-assisted, automated peptide synthesizer. The syntheses were carried out on Tenta Gel R Rink Amide 0.19 mmol/g, or Tenta Gel S Trt 0.25 mmol/g resins from Rapp Polymere GmbH. The amino acids had the 9-fluorenylmethyloxycarbonyl (Fmoc) group for protection of α-amino groups, except for ornithine, which was N^{α}-protected with the 1-(4,4-dimethyl-2,6-dioxocyclohexylidene)ethyl (Dde) group and had Fmoc-protection of the side-chain δ-amino group. Tyrosine, used as a hydrophobic tag for HPLC, was side-chain protected with a *tert*-butyl group. All amino acids and reagents were purchased from Iris Biotech GmbH. Fmoc-D-Glu-OBn was prepared in two steps from Boc-D-Glu-OBn [1]. Racemic *N*,*N*-di-Fmoc-protected 2,6-diaminopimelic acid (*rac*-DAP) was prepared in one step from racemic 2,6-diaminopimelic acid [2]. GlcNAc(β1-4)MurNAc disaccharide was purchased from Toronto Research Chemicals Inc. Peptide couplings were performed using 5 equivalents of amino acid, 5 equivalents of 1-hydroxybenzotriazole (HOBt), 4.75 equivalents of *N*-[(1*H*-benzotriazol-1-yl)(dimethylamino)methylene]-*N*-methylmethanaminium hexafluorophosphate *N*-oxide (HBTU), and 9.75 equivalents of *N*,*N*-diisopropylethylamine (DIEA) in *N*-methylpyrrolidone (NMP). A coupling time of 10 minutes at 75 °C, and subsequent deprotection with piperidine in *N*,*N*-dimethylformamide (DMF) (2:3) for 3 min, followed by piperidine-DMF (1:4) for 10 minutes was used. After each coupling and deprotection step, a washing procedure with 3×NMP, 1×dichloromethane, then 3×NMP was used. Peptides were cleaved from the resin with trifluoroacetic acid-water-triethylsilane 95:3:2 for half an hour and then for two hours. Purification was performed by reverse phase HPLC (Dionex Ultimate 3000 system) on preparative C18 columns (FeF Chemicals, 300 Å, 5 µm, C18 particles, 21.5×250 mm) using a linear gradient flow of water-acetonitrile containing 0.1% TFA. Peptide purity was evaluated by analytical HPLC (Dionex Ultimate 3000 system)

on an analytical C18 column (Phenomenex Gemini NX 110 Å, 5 µm, C18 particles, 4.60×50 mm) using a linear gradient flow of water-acetonitrile containing 0.1% formic acid, and identification was carried out by electrospray ionization mass spectrometry (ESI-MS) (MSQ Plus Mass Spectrometer, Thermo).

S1.1.1. H-L-Ala-γ-D-Glu-L-Lys-D-Ala-D-Ala-OH (fragment 1)

This material was purchased from Sigma-Aldrich Denmark. MS (ESI): calculated for $C_{20}H_{36}N_6O_8$ $[M+H]^+$: 489.27, and $[M+Na]^+$: 511.25; found: m/z 489.3, and 511.3.

S1.1.2. GlcNAc(β1-4)MurNAc(3'-[-L-Ala-γ-D-Glu-α-*ambo*-DAP($N^ε$-Ac)-D-Ala-NH$_2$]) (fragment 2)

On a Rink Amide resin was coupled D-Ala-OH, followed by racemic *N,N*-di-Fmoc-protected 2,6-diaminopimelic acid. After removal of the two Fmoc groups, the resin was treated with a saturated solution of basic copper(II) carbonate in tetrahydrofuran-water 1:1 for 16 h to selectively protect the ε-amino and ε-carboxylic groups by complexation. After washing with tetrahydrofuran-water 1:1, the α-amino group of DAP was Fmoc-protected by treatment with 1.0 equivalent of Fmoc-*O*-succinimidyl ester. Dissociation of the copper complex was achieved by washing the resin with a saturated solution of ethylenediaminetetraacetic acid (EDTA) in tetrahydrofuran-water 1:1 for 16 h, during which a color change of the resin from green to colorless was observed. The ε-amino group of DAP was then acetylated by treatment with acetic anhydride, followed by benzylation of the ε-carboxylic group of DAP with 3 equivalents of benzyl bromide and 1 equivalent of DIEA in DMF for 20 min at 80 °C. After removal of the $N^α$-Fmoc group of DAP, coupling of Fmoc-D-Glu-OBn through the glutamic acid side-chain was followed by deprotection and coupling with Fmoc-L-Ala-OH. After removal of the *N*-terminal Fmoc group, the peptide was cleaved from the resin, and purified by HPLC. MS (ESI): calculated for $C_{34}H_{46}N_6O_9$ $[M+H]^+$: 683.34; found: m/z 682.8. This peptide was coupled in solution with 0.5 equivalents of the unprotected GlcNAc(β1-4)MurNAc disaccharide, and the product was purified by HPLC. MS (ESI): calculated for $C_{53}H_{76}N_8O_{21}$ $[M+H]^+$: 1161.52, and $[M+2H]^{2+}$: 581.27; found: m/z 1161.1, and 580.9. Finally,

the two benzyl groups were removed by treatment with hydrogen in the presence of 10% palladium on charcoal in methanol to provide, after filtration and concentration, the pure product. MS (ESI): calculated for $C_{39}H_{64}N_8O_{21}$ $[M+H]^+$: 981.43, and $[M+Na]^+$: 1003.41; found: m/z 981.5, and 1003.4.

S1.1.3. Ac-L-Ala-γ-D-Glu-L-Orn(*N*^δ-Ac)-D-Ala-D-Ala-L-Tyr-NH$_2$ (fragment 3)

On a Rink Amide resin was coupled Fmoc-L-Tyr(tBu)-OH, Fmoc-D-Ala-OH, Fmoc-D-Ala-OH, and Dde-L-Orn(Fmoc)-OH. After removal of Fmoc from the side-chain of ornithine, the δ-amino group was acetylated. The N^α-Dde group was removed by treatment with 1% hydrazine hydrate in DMF for 2×4 h. Subsequently was coupled Fmoc-D-Glu-OBn through the glutamic acid side-chain, followed by Fmoc-L-Ala-OH. After acetylation (acetic anhydride) of the *N*-terminal amino group, the peptide was cleaved from the resin. Finally, the benzyl group was removed by treatment with hydrogen in the presence of 10% palladium on charcoal in methanol to provide, after filtration and concentration, the pure product. MS (ESI): calculated for $C_{32}H_{48}N_8O_{11}$ $[M+H]^+$: 721.35, and $[M+Na]^+$: 743.33; found: m/z 721.1, and 743.4.

S1.1.4. H-L-Tyr-L-Ala-γ-D-Glu-L-[*N*^δ-(H-L-Tyr-L-Ala-γ-D-Glu-L-Orn(*N*^δ-Ac)-D-Ala-Gly-Gly-)]Orn-D-Ala-D-Ala-OH (fragment 4)

On a Trityl resin was coupled Fmoc-D-Ala-OH, Fmoc-D-Ala-OH, and Dde-L-Orn(Fmoc)-OH. After removal of Fmoc from the side-chain of ornithine, coupling on the δ-amino group was continued with Fmoc-Gly-OH, Fmoc-Gly-OH, Fmoc-D-Ala-OH, and Dde-L-Orn(Fmoc)-OH. After removal of Fmoc from the side-chain of the second ornithine, the δ-amino group was acetylated (acetic anhydride). The two N^α-Dde groups were removed by treatment with 1% hydrazine hydrate in DMF for 2×4 h. Subsequently was coupled Fmoc-D-Glu-OBn through the glutamic acid side-chains, followed by Fmoc-L-Ala-OH, and Fmoc-L-Tyr-OH. After deprotection of the two *N*-terminal amino groups, the peptide was cleaved from the resin, and purified by HPLC. MS (ESI): calculated for $C_{73}H_{99}N_{15}O_{21}$ $[M+H]^+$: 1522.72, and $[M+2H]^{2+}$: m/z 762.87; found: m/z 1522.8, and 762.2. Finally, the two benzyl groups were

removed by treatment with hydrogen in the presence of 10% palladium on charcoal in methanol to provide, after filtration and purification by HPLC, the pure product. MS (ESI): calculated for $C_{59}H_{87}N_{15}O_{21}$ $[M+H]^{+}$: 1342.63, and $[M+2H]^{2+}$: m/z 671.82; found: m/z 1342.9, and 672.2.

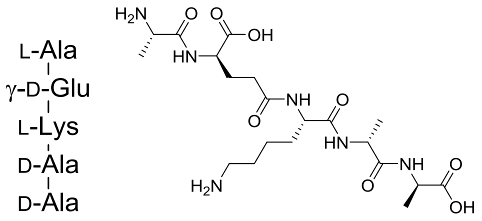

Fragment 1 (Lys-type pentapeptide)

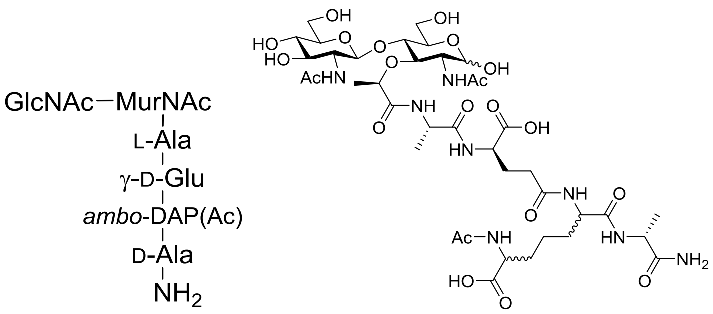

Fragment 2 (DAP-type tetrapeptide disaccharide)

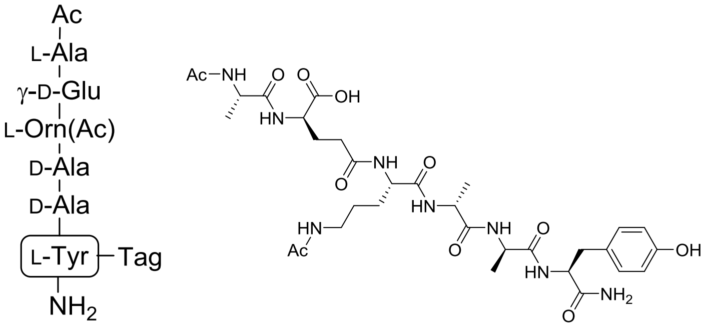

Fragment 3 (tagged *T. thermophilus* pentapeptide)

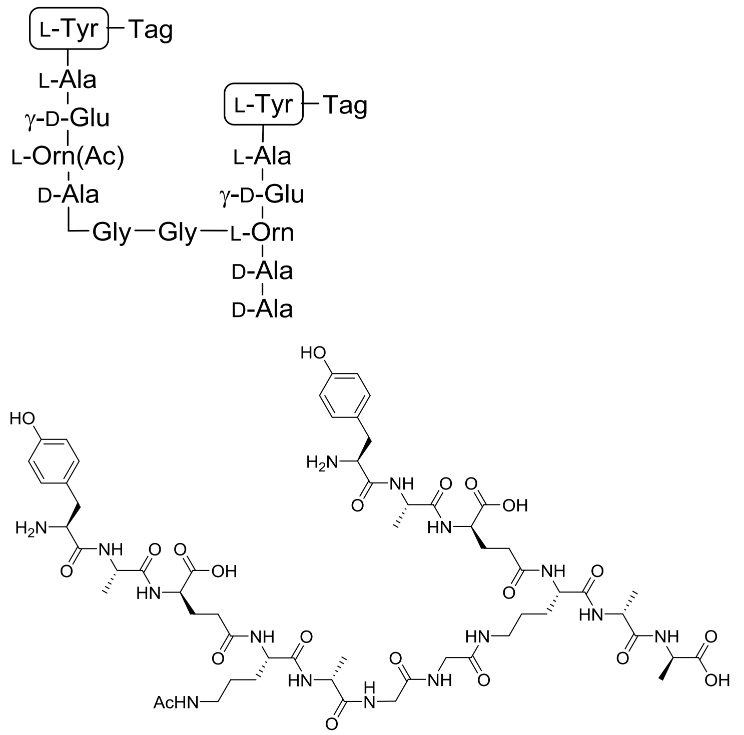

Fragment 4 (tagged *T. thermophilus* cross-linked peptide)

S1.2. LC-MS analysis of incubation mixtures containing peptidoglycan fragments:

Enzyme activity was investigated by LC-MS analysis of incubation mixtures of the protein with peptidoglycan fragments 1-4, essentially in accordance with Kumar et al. [3]. Briefly, 200 μg of peptidoglycan fragment was incubated with 100 μL of the protein at 1 mg /mL in 100 mM phosphate buffer or tris buffer pH 7.0 at 37 °C or 70 °C for 4-16 h. Reactions were terminated by filtration at 12,500 rev/min of the protein on 10 kDa Amicon Ultra centrifugal filter units (Millipore). The filtrate was injected on an analytical LC-MS analysis (Dionex Ultimate 3000 system) on a C18 column (Phenomenex Gemini NX 110 Å, 5 μm, C18 particles, 4.60×50 mm) using water-acetonitrile containing 0.1% formic acid as the mobile phase. A linear gradient flow of 5-30% acetonitrile in water was performed over 5 min. The LC was coupled to MS detection by electrospray ionization mass spectrometry (ESI-MS) (MSQ Plus Mass Spectrometer, Thermo). Negative controls in the form of peptidoglycan fragments in the absence of protein, as well as protein in the absence of peptidoglycan fragment were included. The addition of tyrosines to fragments 3 and 4, in the *C*- and *N*-

terminus, respectively, was intended as a hydrophobic tag to aid separation on reverse phase HPLC.

References

[1] Agnihotri, G.; Ukani, R.; Malladi, S. S.; Warshakoon, H. J.; Balakrishna, R.; Wang, X.; David, S. A. *J. Med. Chem.* **2011**, *54*, 1490-1510

[2] Kok, W. M.; Scanlon, D. B.; Karas, J. A.; Miles, L. A.; Tew, D. J.; Parker, M. W.; Barnham, K. J.; Hutton, C. A. *Chem. Commun.* **2009**, *41*, 6228-6230

[3] Kumar, A.; Kumar, S.; Kumar, D.; Mishra, A.; Dewangan, R. P.; Shrivastava, P.; Ramachandran, S.; Taneja, B. *Acta Cryst. D* **2013**, *D69*, 2543-2554

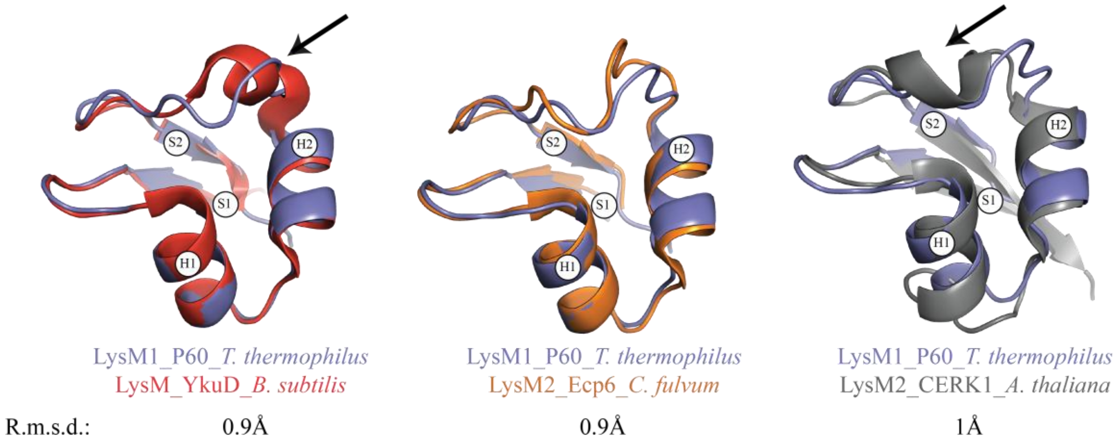

Figure S1 Comparison of P60_tth LysM1 domain to bacterial, fungal, and plant LysM domains. The arrows indicate the main differences observed between P60_tth and the other three structures: Ykud, Ecp6 and CERK1 (PDB accessions: 4A1K, 4B8V and 4EBY respectively).

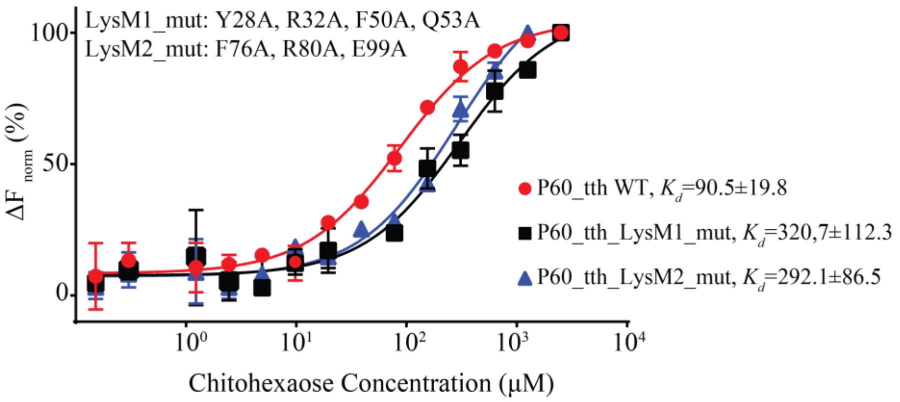

Figure S2 Chitohexaose binding studies by Microscale Thermophoresis. The graph indicates the fit of the data. The y-axis represents the fluorescence expressed in arbitrary units and the x-axis the chitohexaose concentration. The residues mutated in the two LysM domains are those whose side chains are involved in contact with chitohexaose. The dissociation constant K_d are indicated on the figure.
